# Foreword to the focus issue: researches on materials innovation program in University of Tsukuba

**DOI:** 10.1080/14686996.2026.2744053

**Published:** 2026-10-12

**Authors:** Yohei Yamamoto, Takahiro Kondo

**Affiliations:** Department of Materials Science, and Tsukuba Research Center for Energy Materials Science (TREMS), Institute of Pure and Applied Sciences, and Tsukuba Institute for Advanced Research (TIAR), University of Tsukuba, Tsukuba, Ibaraki, Japan

Materials innovation depends on our ability to connect an understanding of matter at the molecular and atomic levels with the design and realization of useful functions. Addressing challenges in energy, environment, information technology, and biotechnology requires close interaction between chemistry, physics, and engineering. Equally important is an educational environment in which young researchers can learn across these disciplines and contribute to research through international collaboration. This Collection, ‘Researches on Materials Innovation Program in University of Tsukuba’, brings together contributions from faculty members of the Materials Innovation Program and their collaborators, illustrating the breadth of research that supports this shared endeavor.

The Materials Innovation Degree Program at University of Tsukuba was established in April 2020 as part of the university’s reorganization of graduate education into a degree program system. Offering master’s and doctoral courses, the program aims to develop researchers who can create new materials and technologies and contribute internationally to their practical implementation. Its education is built on three complementary pillars: materials design, materials analysis, and materials synthesis and device fabrication. Lectures and seminars are conducted in English, bringing students from different backgrounds into a common research environment. The program also draws on the concentration of research expertise and advanced facilities in Tsukuba, including the National Institute for Materials Science (NIMS), the National Institute of Advanced Industrial Science and Technology (AIST), and the High Energy Accelerator Research Organization (KEK), while encouraging interaction with industry.

This commitment to international collaboration is also reflected in the program’s continued co-organization of the Southeast Asia Collaborative Symposium on Energy Materials (SACSEM) with partner institutions. The symposium series began in Tsukuba in 2018, preceding the establishment of the current degree program, and reached its 11th edition in Malaysia in October 2025. Through meetings in Japan and Southeast Asia, as well as online events, SACSEM provides a regular forum for researchers and students to discuss advances in energy materials. Student oral and poster presentations offer opportunities to communicate research findings, exchange ideas with international participants, and build relationships across institutions and countries.

The nine contributions in this Collection span molecular dynamics, biomolecular interfaces, hydrogen storage, carbon nanomaterials, magnetic thin films, chiral polymers, optics, and electrocatalysis. Across these diverse topics, a recurring objective is to understand how molecular interactions, structural organization, and processing conditions determine materials properties and performance.

At the interface of molecular science and biology, Murakami et al. investigate the reorientational dynamics of amino acids in aqueous solution using ultrafast optical Kerr effect spectroscopy [1]. Their comparison with a polymer solution highlights the distinction between the local rotational environment experienced by solutes and macroscopic viscosity, demonstrating the value of spectroscopy as a complement to rheological measurements. Focusing on biomolecular interactions with solid surfaces, Kanoh et al. review protein and peptide separation using inorganic oxides [2]. They relate adsorption mechanisms to surface chemistry and material structure, providing guidance for the selection and engineering of materials for selective and mild purification processes.

Molecular design and structural organization also underpin the optical functions highlighted in this Collection. Yamamoto and Kushida discuss ‘optochemistry’ as an intersection of chemistry and optics that emphasizes the wave and beam characteristics of light [3]. Their review connects the properties and self-assembled structures of organic molecules and polymers with optical resonators, lasers, and light–matter strong coupling. Suzuki et al. demonstrate how controlling aggregate formation in chiral thienopyrroledione–fluorene π-conjugated polymers enables reproducible circular dichroism and circularly polarized luminescence [4]. Their findings emphasize that preparation protocols are integral to obtaining reliable optical functionality from molecular assemblies.

Advanced characterization and computational approaches provide further routes to materials innovation. Tang et al. introduce ‘VibeTube’, an open-source, physics-based Python code for extracting carbon nanotube chiralities from transmission electron microscopy images [5]. Developed with assistance from an artificial intelligence coding tool, the method combines accurate diameter measurements with analysis of Fourier-transform features. In magnetic recording materials, Ogawa et al. integrate experiments with machine learning and Bayesian optimization to control the microstructure and magnetic properties of FePt–BN granular films for heat-assisted magnetic recording [6]. Complementing this work, Yasuda and Suemasu survey room-temperature magnetic compensation in impurity-doped Mn_4_N epitaxial films and discuss thin-film structures relevant to their integration into spintronic devices [7]. Together, these studies illustrate how quantitative structural analysis and control over composition and processing can guide the development of functional nanomaterials.

Energy-related materials form another important strand of the Collection. Oki and Kondo review UiO-type metal–organic frameworks as platforms for hydrogen storage studies [8]. They discuss the roles of pore structure, functionalization, and densification in physisorption, alongside approaches involving hydrogen spillover and the nanoconfinement of hydrides. Turning to electrochemical energy conversion, Tahawy et al. examine how the functional groups of organic energy carriers influence catalyst poisoning on non-noble high-entropy alloy anodes in acidic media [9]. Their study links molecular reactivity and surface-blocking polymer formation to electrode degradation, offering insight into the design of more durable electrochemical systems.

Taken together, these contributions reflect an approach to materials innovation in which fundamental understanding, precise characterization, and functional materials development inform one another. They also illustrate the range of research opportunities available in an international graduate program connecting university laboratories, national research institutes, and industrial partners. We hope that this Collection will encourage readers to explore connections across materials research fields and stimulate new collaborations, while inspiring young researchers to develop their own contributions to materials science and technology.

We sincerely thank all authors for contributing their work to this Collection, the reviewers for their constructive assessments, and the editorial team of Science and Technology of Advanced Materials for their support in bringing the Collection to publication.

## References

[1] Murakami K, Nobeyama T, Koda Y, et al. Reorientational dynamics of amino acids in aqueous solution probed by ultrafast optical kerr effect spectroscopy. Sci Technol Adv Mater. 2026. doi: 10.1080/14686996.2026.2736324

[2] Kanoh S, Shiraki K, Kato K, et al. Protein separation and purification using inorganic oxide materials: from surface chemistry to biochemical applications. Sci Technol Adv Mater. 2026;27(1):2701640. doi: 10.1080/14686996.2026.2701640 PMC13577285 42746599

[3] Yamamoto Y, Kushida S. “Optochemistry”, when chemistry meets optics. Sci Technol Adv Mater. 2026;27(1):2665926. doi: 10.1080/14686996.2026.2665926 PMC13218310 42222276

[4] Suzuki N, Hu Z, Nakayama S, et al. Reproducible chiroptical activity from aggregated chiral thienopyrroledione–fluorene π-conjugated polymers. Sci Technol Adv Mater. 2026;27(1):2680968. doi: 10.1080/14686996.2026.2680968 PMC13292314 42358809

[5] Tang D, Raj G, Picheau E, et al. VibeTube: a code for accurate extraction of CNTs’ chirality from TEM images. Sci Technol Adv Mater. 2026;27(1):2705816. doi: 10.1080/14686996.2026.2705816 PMC13495031 42630665

[6] Ogawa D, Shoji T, Yano M, et al. Machine learning-driven analysis and process optimization of FePt-BN granular films for HAMR applications. Sci Technol Adv Mater. 2026;27(1). doi: 10.1080/14686996.2026.2699076 PMC13629823 42824899

[7] Yasuda T, Suemasu T. Current status of room temperature magnetic compensation in impurity-doped Mn_4_N epitaxial thin films. Sci Technol Adv Mater. 2026;27(1):2688056. doi: 10.1080/14686996.2026.2688056 PMC13292317 42358811

[8] Oki O, Kondo T. UiO-type metal–organic frameworks as a platform for hydrogen storage studies. Sci Technol Adv Mater. 2026;27(1):2698228. doi: 10.1080/14686996.2026.2698228 PMC13528131 42676817

[9] Tahawy R, Muflihah SA, Hara K, et al. Catalyst poisoning influences from various functional groups of energy carriers towards electrochemical oxidation reactions on non-noble high-entropy alloy anodes in acidic media. Sci Technol Adv Mater. 2026;27(1):2653417. doi: 10.1080/14686996.2026.2653417 PMC13148096 42100153

